# Indents

**Published:** 1915-04

**Authors:** 


### INDENTS.

☞ **Brief Articles of Technical or Scientific Value will receive notice and comment. Contributions invited.**

*The Employment of Aluminum Dentures and the Attachment of Artificial Teeth and Gum to Cast Plates.*

When aluminum was used for casting purposes, as early as 1888, the metal was not as pure as it can be obtained today, and Carroll, who reported his system of casting aluminum under compressed air in the DENTAL REGISTER, added platinum, silver, and copper to overcome the considerable contraction of the impure metal at his command. Owing to the low specific gravity of aluminum, which when cast is 2.64, when rolled 2.68, when drawn 2.70, it is necessary to exert a continuous pressure when casting in order to obtain perfect adaptation to the model.

Wünsche briefly reviews the history and metallurgy of aluminum, which today is obtained 99¾ per cent. pure. In casting, the contraction, in his opinion, is best overcome by the use of Wood's centrifugal casting apparatus, in which the pressure is uniformly maintained during the transition of the metal from the molten to the solid state, thereby insuring a close adaptation of the plate and avoiding porosity. The slight expansion of the plaster impression and the cast is counteracted by the slight contraction of the aluminum in casting. In taking the impression of the mouth, all hard portions must be carefully noted and relieved, in order to avoid pressure or rocking in the finished plate. The cast is poured in investment compound, and the plate, as well as the channels connecting it with the three sprue holes in the casting flask, is modeled in wax, which is coated with thickly mixed investment compound, and invested while on the model. After slow, gradual drying, the wax is carefully burned out, making sure that no wax vapor remains in the flask. After the molten metal has been poured into the flask, the centrifugal machine is kept in steady motion for three minutes. Wünsche employs continuous-gum work on his cast aluminum plates, building up the gum in wax on a model of investment compound, and pouring counter-dies of plaster, to be used in pressing the porcelain body after the wax has been burned out. Instead of investment compound and plaster, synthetic stone may be advantageously used for making this die and counter-dies. The porcelain body is baked to a bisque and covered with pink enamel of a lower fusing-point. The continuous-gum work is either cemented or vulcanized to the cast aluminum plate. In lower plates a lug of porcelain on either side buccally will greatly aid in retention in cases of advanced absorption of the alveolar ridge.

Wünsche claims great hygienic as well as anatomic advantages for this method. He warns against the use of hydrogen dioxid in mouth-washes, etc., in connection with aluminum prostheses. Excessive smokers sometimes complain of an insipid taste, which they attribute to the aluminum, but which may usually be ascribed to negligence in cleaning the plate.—Dr. E. Wünsche, in *Cosmos* for January.

*Gold Filling vs. Gold Inlay.* The only question for us to settle is whether the inlay is as good a tooth saver as the gold filling. In answering that question the personal equation plays so great a part that it is difficult to arbitrarily say that a gold inlay will save a tooth as well as a gold filling, for we may compare a good foil filling with a poor inlay or vice versa. I can say, however, without fear of successful contradiction, that a poor inlay is vastly superior to a poor gold filling, for the cement lining will preserve the tooth for a long period of time even though the inlay does not fit the cavity; indeed, I have seen inlays that did not seem to have been made for the cavity in which they were placed, so great was the discrepancy between the inlay and the tooth, and yet there was no decay around the margins. I have two such inlays under observation now that have been in the teeth for four or five years and though there have been other cavities in the patients' teeth, showing that there was a susceptibility to decay, there was no decay around these imperfect inlays. If a poorly made gold filling is placed in a tooth the leakage commences immediately and failure begins as soon as the rubber dam is removed from the tooth.—J. V. Conzett, in *Review*.

*Applying Desensitizing Paste to Live Teeth Preparatory to Crowning.*

Separation is secured with separating discs and the lingual and buccal plates of enamel are ground away, also the enamel of the occlusal surface. The object being to get as complete an exposure of dentin as possible, particularly so in the regions requiring additional grinding. The enamel may be ground away without pain or discomfort to the patient.

A suitable celluloid tooth form or shell is next selected and trimmed to approximate the gingival margin of the tooth, after which it is placed in hot water to soften.

The tooth is now protected with cotton rolls and thoroughly dried, and the Desensitizing paste applied to all areas requiring additional grinding. For the purpose of sealing the celluloid cap in position either a temporary cement, a quickly evaporating cavity lining or sandarac may be used. I believe sandarac varnish to be the most satisfactory. The tooth is covered with sandarac and the softened celluloid cap pressed to position—doubling the celluloid upon itself lingually and buccally if necessary to get close adaptation in the gingival third. While still soft the patient closes to secure proper occlusion, after which the form is chilled to harden. This will seal perfectly for forty-eight hours or longer if necessary.—J. H. Harrison, in *Dental Review*.

*Pain Caused by Denture.* When it comes to the question of artificial dentures, I think the average dentist seldom thinks of the amount of pain he may cause by a lack of judgment in adjusting them. I speak particularly of full dentures. I think ,everyone is conscious of the fact that he has had in his own practice this experience. The edentulous patient will complain of the same kind of pain he had before his teeth were extracted if he wears artificial dentures. He feels comfortable when the dentures are out, but when he puts the denture in he has the same old toothache which he had before. Really there is no mystery about these pains. It is like the mysterious neuralgias which existed prior to the advent of Roentgen photography. Patients were given quinin and morphin and everything under the sun to control pain, so much so that many patients acquired the morphin habit, due to the administration of morphin for the control of pain, but which pain had its origin from a specific source which was never discovered and so the physicians assumed the pain was caused by a general systemic disturbance. The pain which is complained of by the edentulous patient is due to the pressure of an ill-advised plate, which is constructed on an ill-advised plan. The dentist who makes a plate oftentimes forgets to take into consideration the anatomy of the part; he takes an impression, makes his cast and plate, and puts it in, and the patient has so much pain that it is a sense of relief to take the plate out. The pain is due to pressure upon the terminal branches of the fifth nerve. If he makes a plate without considering the points of exit of these nerves, makes the same pressure upon these nerves that he does elsewhere, it would be just like making a saddle to put on a horse's back without considering the animal's spine. It would produce such an abrasion of the spine that the horse could not bear it. And so it is with a plate. A plate should be so constructed as to make little depressions in it to fit over the nerve termini like the saddle on a horse's back. When it comes to a lower denture, especially in the very aged, in whose cases the mental foramina are on the summit of the alveolar border, when you put your finger in the mouth of such a patient you can feel a distinct ridge that has a nerve that emerges from that point. The average dentist in making a denture does not consider the anatomical peculiarity of the fit, but when the denture is put in it creates such pressure that it causes pain. There is no mystery about such a pain. It is a lack of judgment or thoughtlessness on the part of the dentist.—T. W. Brophy, in *Review*.

*The Treatment of Sensitive Cavities.* The fear of dentistry and the need of more gentle and painless methods of treating sensitive cavities have brought about new methods of doing so. Analgesia by $N_2O$ and O is neither convenient nor necessary. The ethyl chlorid spray is very useful when immediate treatment is required.

Hypodermic injections are also very useful in certain cases.

The use of formaldehyd gas given off by paraform has the useful properties of hardening gelatin, rendering it insoluble and inflammable; it works very quickly in the teeth of young people and on carious dentin; it is very irritating if used near the pulp, therefore a good deal of judgment is required. The use of para-mono-chloro-phenol, a preparation of carbolic acid and chlorin, is non-irritating and strongly antiseptic and anodyne; it is used in cases where the caries has penetrated near the pulp. If a tooth has given sufficient pain to keep the patient awake at night it should be devitalized. The para-mono-chloro-phenol has a modifying effect on the arsenic, greatly lessening pain. The dentin of young people is much more porous than that of old dentinal tubes obliterated in old subjects—C. E. Brown in January *Cosmos.*

*Removable Bridge Work and Saddle Bridges.* The root-canals must be easy of access, and the root as little weakened as possible. The bridges must be made so that they will fasten well and yet be easy to remove. When the bridge pillars are molars and premolars, they are cut down a little below half height, and the sides are made cylindrical—with a transverse section like that of the root. A cylindrical ring of platinum-iridium or platinum-silver, with a cover of the same material, is made to cover these and soldered to it. Over this another cylindrical platinum-iridium or platinum-silver ring is adjusted. To the exterior of the latter, on the visible side, is soldered a thin gold plate twenty-four karats fine. On the exterior ring the crown cover is cast with a buttonlike stop down to the capsule directly placed over the root. If a greater contour of the exterior capsule is required mesially or distally, a little of the upper edge is removed at this place, and the contour is restored by casting in gold on the ring together with the crown cover. When the bridge pillars are incisors and canines, a thinner platinum-iridium or platinum-silver cap with tube fastened to it—which must not be too substantial—is used. Outside this is adjusted a platinum-iridium or platinum-silver ring a little thicker than the former. On this is soldered a cover and a pin fitting into the tube. A steel facing is now ground on in such a manner that the foremost part of the exterior cap is removed. The tooth is now standing upon the foremost edge of the interior cap, and is soldered to the exterior cap. The remainder of the foremost halfpart of the ring of the exterior cap is then removed. The foremost visible part of the ring of the interior cap is covered with gold twenty-four karats fine.

These appliances were used in—I, Bridges; II, Saddle Bridges; III, In connection with Prosthesis.

EXAMPLES OF I.

(*a*) In the upper jaw, second molar and first premolar remaining in right side; and in the left side, second premolar and first molar. These are prepared with double capsules; upon this is placed a complete removable upper denture without palate.

(*b*) On the root of two canines and on two molars a complete upper denture without palate is fastened by using the different preparations of the different bridge pillars.

EXAMPLES OF II.

(*a*) Molars wanting in the upper or lower half jaw of one side. To the premolars in the same half of the jaw double capsules are adjusted. Such exterior capsules are soldered together and connected with the gold or rubber which is to replace the molars.

(*b*) In the upper jaw the two premolars and the first molar are wanting, second and third molar are prepared in same way as explained in (*a*) preceding, and connected with the gold or rubber which is to replace the teeth.

(*c*) Lower jaw. In one side molars and premolars are wanting. First premolar of the other side is shortened and covered with inside capsule. The similar outside capsule is with a bar connected with a double capsule with tube and pin of the canine on the other side, and this again connected with the denture replacing the wanting teeth.

EXAMPLES OF III.

(*a*) Lower jaw. Only two second molars, one on each side, are left. All the rest of the teeth are wanting. Double capsules. The exterior ones are connected with the denture.

(*b*) In the upper jaw are only left two incisors or canines. Double capsules with tubes and pins, the exterior ones of which are connected with an upper denture. —By A. C. S. Angel in *Cosmos*.

